# Application of “Galileo High Accuracy Service” on Single-Point Positioning

**DOI:** 10.3390/s23094223

**Published:** 2023-04-23

**Authors:** Antonio Angrisano, Silvia Ascione, Giovanni Cappello, Ciro Gioia, Salvatore Gaglione

**Affiliations:** 1 Department of Engineering, Messina University, 98166 Messina, Italy; antonio.angrisano@unime.it; 2 International PhD Programme “Environmental Phenomena and Risks (EnPheR)”, Department of Science and Technology, “Parthenope” University of Naples, 80143 Napoli, Italy; silvia.ascione001@studenti.uniparthenope.it; 3 International PhD Programme UNESCO Chair “Environment, Resources and Sustainable Development”, Department of Science and Technology, “Parthenope” University of Naples, 80143 Napoli, Italy; salvatore.gaglione@uniparthenope.it; 4 Independent Researcher, 21020 Brebbia, Italy; cirogioia@tin.it; 5 Department of Science and Technology, “Parthenope” University of Naples, 80143 Napoli, Italy

**Keywords:** High-Accuracy Service (HAS), GNSS, Single-Point Positioning, GPS, Galileo

## Abstract

Employment of precise positioning techniques will enable low-cost receivers for a variety of applications. The complexity of techniques such as Precise Point Positioning (PPP), or differential techniques that require the use of external sources of corrections, could be a disadvantage for users. On the other hand, a simple technique such as Single-Point Positioning (SPP) alone does not provide high-level accuracy. Nevertheless, the entry Galileo High-Accuracy Service (HAS), even if developed to be applied on PPP, could offer a positive impact on SPP. The objective of this study is the analysis of the effects of HAS on SPP, which are evaluated for Galileo and GPS, in single- and double-constellation mode. Results are encouraging, especially on the vertical channel, where some centimetric improvements are obtained.

## 1. Introduction

The accuracy of the Global Navigation Satellite System (GNSS) is an important aspect to evaluate the performance of navigation algorithms, and it is constantly improving thanks to the use of new techniques or services. In the satellite navigation field, several positioning methods could be used, differing from each other in the algorithm complexity, the need for external infrastructures (such as differential techniques), or the need for external information. The simplest technique is Single-Point Positioning (SPP), which can provide metric-order solutions [1].

Since 2023, a new open-access service, named the High-Accuracy Service (HAS), has been introduced by Galileo, with the aim of achieving a decimeter level of accuracy. HAS provides corrections for GPS and Galileo systems and is designed to be used in a Precise Point Positioning (PPP) algorithm. The corrections include orbits, satellite clocks, and code and carrier phase biases [2], though the latter is not yet available [3]. The corrections are transmitted on the E6 signal of Galileo [2,4] and are disseminated by the internet as well [5]. HAS will increase the performance of the navigation solution, leading to benefits in different fields, such as aerospace, maritime, and automotive [6,7]. The EU Agency for the Space Programme (EUSPA) declared the operability of Galileo HAS services in January 2023 [7]. In metrics terms, the expected positioning performance with HAS is about 20 and 40 cm on horizontal and vertical components, respectively [6], with a confidence level of 95%. HAS supports Galileo and GPS constellations, respectively, on E1, E5a, E5b, E6, E5AltBOC, and L1, L5, and L2C signal frequencies.

Galileo HAS has been the subject of interest of several research groups. In [8], a comparative analysis on the performance levels, using data from worldwide stations, regarding the quality of the transmitted correction, is realized. In [9], the benefits of HAS in terms of coverage and accuracy are shown. The authors of [10] rigorously described the initial history of the service, its architecture, and its benefits. In [11], an open-source library developed in Python is presented and described, and the tool is able to decode the HAS corrections. In [12], live HAS corrections are analyzed in the presence of a Galileo satellite clock anomaly. This study showed that thanks to HAS corrections, a user could mitigate the effect of satellite faults even if the HAS message does not include integrity information. An analysis of the benefits of HAS on real-time orbit determination of Low Earth Orbit (LEO) satellites is carried out in [13], and the results demonstrate the high potentialities of this service. In [14], a decoder of HAS corrections transmitted through the Galileo E6B signal is described, and then the effects of them on the SPP technique are evaluated on E1, E5a, and E5b.

Galileo HAS has been developed for precise positioning using Galileo and GPS. Currently, different GNSS are developing (or have developed) a similar service. For example, QZSS (Quasi-Zenith Satellite System) has MADOCA-PPP (Multi-GNSS ADvanced Orbit and Clock Augmentation—Precise Point Positioning), and the service provides corrections for GPS, GLONASS, and Galileo. The trial service phase started on 30 September 2022 [15]. Similar to the Galileo HAS, it provides code, phase, orbit, and clock corrections for PPP applications through the L6 signal and, in addition, wide-area ionospheric correction for the Asia–Oceania region [16].

These services are designed for PPP application, exploiting carrier phase measurements. On the other hand, SPP provides a less accurate position solution, but it is independent from external sources or products and does not suffer the drawback of the convergence time or the typical problems related to the use of carrier phase observations.

The purpose of this study is to evaluate the benefits of HAS corrections on SPP for Galileo E1, E5a, E5b, and E6, for GPS L1 and L2, and for GPS/Galileo L1/E1. A comparison between different grade devices is carried out, too. Indeed, the analysis is carried out using a low-cost multi-GNSS receiver and a geodetic multi-GNSS receiver mounted in a permanent station. The effects of HAS on SPP are evaluated in terms of mean and root mean square (RMS) errors in horizontal, vertical, and 3D positions.

The paper is organized as follows: Section 2 describes the combination of the SPP technique and HAS corrections, showing how the corrections are applied on that technique. Section 3 describes the test setup and Section 4 discusses the results; finally, Section 5 concludes the paper.

## 2. Single-Point Positioning and HAS Corrections Application

**Figure 1:**
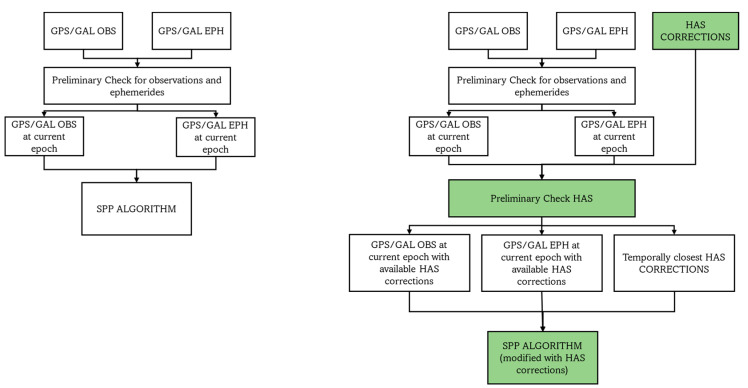
Comparison between a classical SPP algorithm (**left**) and a customized SPP algorithm (**right**), until the preliminary check step.

SPP is a code-based technique largely employed in the satellite navigation field. Even though SPP accuracy is now overtaken by other techniques, such as differential or PPP ones, its strength is due to its simplicity of use and independence from external sources of corrections. From this, the idea of investigating the benefit derived from the application of HAS corrections to SPP emerged. Figure 1 shows the differences between the standard SPP algorithm (on the left) and the customized SPP algorithm, including HAS corrections (on the right). The two algorithms have common elements, identified by the white blocks, while the green boxes indicate the additional elements, including the HAS corrections. In both algorithms, the preliminary check step is performed to select the observations and the ephemerides, at the current epoch. For the HAS case, an additional step is introduced to select the available (depending on the validity time of the corrections [2]) HAS corrections. In addition, the block of the PVT computation has been modified to apply the HAS corrections. Further details about the computation of the position with the inclusion of HAS corrections are presented below.

**Figure 2:**
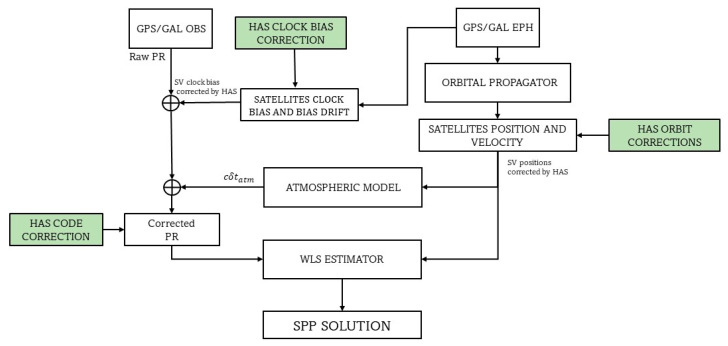
SPP scheme with application of HAS corrections.

Figure 2 shows the proposed approach, that consists in a classical SPP, where, at each epoch, HAS corrections were applied, in order to correct the satellite’s position, satellite clock error, and code bias affecting the measurements.

To be specific, Equation (1) represents the equation of a raw pseudo-range measurement:

(1) $$\rho =\sqrt{{\left(X_{r}-X^{s}\right)}^{2}+{\left(Y_{r}-Y^{s}\right)}^{2}+{\left(Z_{r}-Z^{s}\right)}^{2}}+c\delta t_{r}+c\delta t^{s}-TGD+\delta_{Rel}+\delta_{Sag}+\delta_{i}+\delta_{t}+\delta_{m}+\delta_{h}$$

where, $X_{r},Y_{r},Z_{r}$ are the receiver coordinates, $X^{s},Y^{s},Z^{s}$ are the satellite coordinates, and hence $\sqrt{{\left(X_{r}-X^{s}\right)}^{2}+{\left(Y_{r}-Y^{s}\right)}^{2}+{\left(Z_{r}-Z^{s}\right)}^{2}}$ is the geometrical satellite-receiver distance, *c* is the speed of light, and $\delta t_{r}$ and $\delta t^{s}$ are the receiver and satellite clock errors, respectively. The remaining terms represent the time group delay (TGD), the relativistic error ${(\delta }_{Rel}$), the Sagnac effect ($\delta_{Sag}$), the ionospheric delay ($\delta_{i}$), tropospheric error ($\delta_{t}$), and multipath ($\delta_{m}$) and hardware ($\delta_{h}$) errors [1,17,18].

The relativistic error, the Sagnac effect, the ionospheric delay, and the tropospheric error were corrected using models. In particular, the relativistic effect was corrected using the approach reported in [1,2], the Sagnac effect was corrected according to [1], the ionospheric delay was estimated using the Klobuchar model [19], and the tropospheric error was corrected using the Saastamoinen model [20]. For the standard processing, the TGD was computed using the parameters contained in the navigation message, while for the HAS case, the TGD correction was not applied, as reported in [2].

To this equation, HAS clock corrections were applied in order to correct the term $\delta t^{s}$, while HAS code corrections consisted of an additional term to insert in (1) which corrected the bias characterizing the single measurement. Finally, as clarified by Figure 2, orbital corrections were applied to satellite positions.

The classical approach computes the clock correction using the polynomial model described in [21,22]. The model is based on the broadcast parameter available in the navigation message of the systems. From Equation (2), it can be noted that the refined HAS satellites’ clock corrections include two terms: the first one is computed by the broadcast parameters ($\delta t^{s}$), and the second one is computed by exploiting HAS corrections:

(2) $$\delta t_{HAS}^{s}=\delta t^{s}+\frac{{\delta_{clock}}_{HAS}}{c}$$

where ${\delta_{clock}}_{HAS}$ is computed by multiplying the *DCM* (delta clock multiplier) and the respective *DCC* (delta clock correction), both contained in the HAS corrections dataset [2].

A refined satellite position is obtained by applying HAS orbital corrections to the satellite positions computed from broadcast ephemerides. These corrections (expressed in the SCS (Satellite Coordinate System) NTW reference frame) are contained in the HAS orbital corrections dataset for each satellite, and they are composed by radial (*N*), tangential (also “in-track”, *T*), and normal (also “cross-track”, *W*) components. This vector, indicated as $\underline{\delta R}$, must be rotated from the SCS-NTW to the Earth-Centered Earth-Fixed (ECEF) frame, multiplying it by a rotation matrix, $C_{NTW}^{ECEF}$. Details about the determination of each column of that matrix can be found in [2]. The obtained ${\underline{\delta x}}_{HAS}$ in ECEF was used to correct the ECEF coordinates of the satellites, as shown in (3):

(3) $${\underline{x}}_{HAS}^{s}={\underline{x}}^{s}+{\underline{\delta x}}_{HAS}$$

where ${\underline{x}}^{s}$ is the satellite position computed using only broadcast Keplerian parameters [21,22], and ${\underline{x}}_{HAS}^{s}$ is the refined satellite position corrected through the HAS orbital corrections.

HAS code bias corrections, $\delta \rho_{HAS,k}$, are directly applied to the pseudo-range measurements:

(4) $$\rho_{j,k_{HAS}}=\rho_{j,k}+\delta \rho_{HAS_{j,k}}$$

where the subscripts *j* and *k* indicate the *j*-th satellites and *k*-th signal frequency, respectively.

Equations (2)–(4) and further details about the computation and application of HAS corrections can be found in [2].

For the solution estimation, a set of at least 4 satellites is required if operating in single-constellation mode (or 5 in double-constellation mode). Equation (1) is linearized, and the obtained system of equations can be treated with an estimation technique, which in this work was a weighted least squares (WLS) technique. The solution is obtained as in (5):

(5) $$\underline{\Delta x}={\left(H^{T}W^{-1}H\right)}^{-1}W^{-1}\underline{\Delta \rho }$$

in which, Δ*x* is the vector containing the corrections to update the receiver position and its clock offset. It is composed by four unknown parameters if the positioning is performed with a single GNSS (three components of position *x* and the receiver clock offset) or five unknown parameters if two GNSS systems are employed (the fourth component is the receiver clock offset from the GPS timescale, while the last component is the difference between the two satellite systems’ timescales [23]). In the multi-constellation case, the additional unknown is used to estimate the inter-system bias between GPS and Galileo. The inter-system bias could also be solved by using the Galileo to GPS time Offset (GGTO) parameters available in the Galileo navigation message. The broadcast parameters do not take into account the local delay introduced by the receiver [24], leading to an increased error in the navigation solution [23]. The state vectors of the single- and multi-constellation cases are shown in Equation (6):

(6) $${\underline{\Delta x}}_{SC}=\left[\Delta x_{1}\Delta x_{2}\Delta x_{3}\Delta c\delta t_{r}\right]\;{\underline{\Delta x}}_{MC}=\left[\Delta x_{1}\Delta x_{2}\Delta x_{3}\Delta c\delta t_{r}\Delta c\delta t_{sys}\right]$$

*H* is the design matrix containing, for each satellite, the partial derivative of the pseudo-range measurement with respect to the four or five unknowns [1,25]:

(7) $$H_{SC}=\left[\begin{array}{cccc} a_{x_{1}}^{1} & a_{x_{2}}^{1} & a_{x_{3}}^{1} & 1\\ a_{x_{1}}^{2} & a_{x_{2}}^{2} & a_{x_{3}}^{2} & 1\\ \vdots & \vdots & \vdots & \vdots \\ a_{x_{1}}^{n} & a_{x_{1}}^{n} & a_{x_{1}}^{n} & 1 \end{array}\right]\;H_{MC}=\left[\begin{array}{ccccc} a_{x_{1}}^{g_{1}} & a_{x_{2}}^{g_{1}} & a_{x_{3}}^{g_{1}} & 1 & 0\\ a_{x_{1}}^{g_{2}} & a_{x_{2}}^{g_{2}} & a_{x_{3}}^{g_{2}} & 1 & \vdots \\ \vdots & \vdots & \vdots & 1 & 0\\ a_{x_{1}}^{g_{m}} & a_{x_{2}}^{g_{m}} & a_{x_{3}}^{g_{m}} & 1 & 0\\ a_{x_{1}}^{e_{1}} & a_{x_{2}}^{e_{1}} & a_{x_{3}}^{e_{1}} & 1 & 1\\ a_{x_{1}}^{e_{2}} & a_{x_{2}}^{e_{2}} & a_{x_{3}}^{e_{2}} & 1 & 1\\ \vdots & \vdots & \vdots & \vdots & \vdots \\ a_{x_{1}}^{e_{n}} & a_{x_{2}}^{e_{n}} & a_{x_{3}}^{e_{n}} & 1 & 1 \end{array}\right]$$

The superscripts *g* and *e* refer to GPS and Galileo satellites, respectively.

Δ*ρ*, defined as a measurement vector, contains the difference between the predicted and corrected pseudo-range.

*W* is the weighting matrix, and its diagonal elements contain the inverse of the variance (*σ_j_^2^*) of each measurement. In particular, the variance values depend on the adopted weighting technique, aimed to provide a different level of importance to each measure [1]. In this study, an elevation-based technique was used, and it is shown in (8):

(8) $$\sigma_{j}^{2}=\frac{1}{\sin^{2}\left(h_{j}\right)}$$

where *h_j_* represents the elevation of the *j*-th satellite.

## 3. Test Setup

In this study, about four hours of data were considered. The data were collected using a professional and a mass-market receiver. The professional receiver data have been collected at the “University of Padova, Center for Space” permanent station (acquired by a STONEX SC2200 professional receiver manufactured by STONEX (Paderno Dugnano, Italy)), whose satellite data and information can be found on the BKG GNSS Data Center website [26]. The receiver was connected to a STXSA1500 multi-GNSS (GPS, Glonass, Galileo, Beidou, IRNSS—Indian Regional Navigation Satellite System) and a multi-frequency antenna. This setup can provide measurements on E1, E5a, E5b, E5, and E6 for Galileo and L1, L2, and L5 for GPS [26,27,28]. A different use case has been considered using a mass-market receiver, a U-Blox F9P. It is also multi-constellation (GPS, Glonass, Galileo, Beidou) and multi-frequency, being able to acquire E1 and E5b for Galileo and L1 and L2 for GPS [29]. As specified in Section 1, HAS corrections are accessible in two ways: through the E6-B signal (for those receivers able to acquire that specific signal) or via the internet [7]. It is possible to request the HAS Internet Data Distribution through Reference [30]. For this study, HAS corrections have been obtained by using the live signal and the decoder, as described in [14].

In this study, only Galileo and GPS measurements were considered, and the frequencies tracked by the two devices were: E1 and E5b for Galileo, and L1 and L2 for GPS for the low-cost receiver, and E1, E5a, E5b, and E6 for Galileo, and L1 and L2 for GPS for the professional receiver.

**Figure 3:**
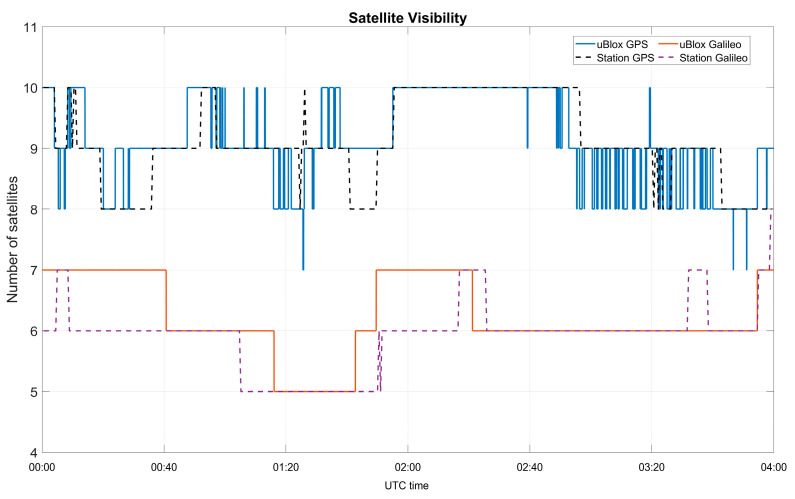
Number of visible satellites as a function of the time, for the U-Blox (continuous lines) and the professional receiver (dashed lines).

Both datasets were collected in open-sky conditions, with a high satellite visibility. The number of visible satellites during the test is shown in Figure 3. From the figure, only small differences can be noted between the two devices. In this study, a satellite was considered visible when its elevation was higher than 5 degrees and the C/N0 was higher than 20 dB-Hz.

**Figure 4:**
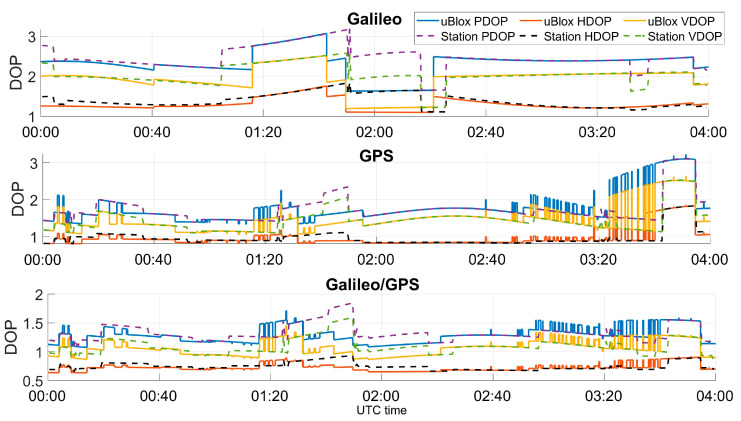
DOP evolution at the two locations of the test, for the single- and multi-constellation cases.

The open-sky conditions were also confirmed by analyzing the dilution of precision (DOP) values. Horizontal DOP (HDOP), vertical DOP (VDOP), and position DOP (PDOP) are plotted as a function of time in Figure 4. In the upper and central boxes, the single-constellation cases are shown, and HDOP varied between 1 and 2 for Galileo and between 0.5 and 1.5 for GPS. The geometry conditions were improved in the multi-constellation case; in this case, the HDOP values were in the range [0.5, 1].

**Figure 5:**
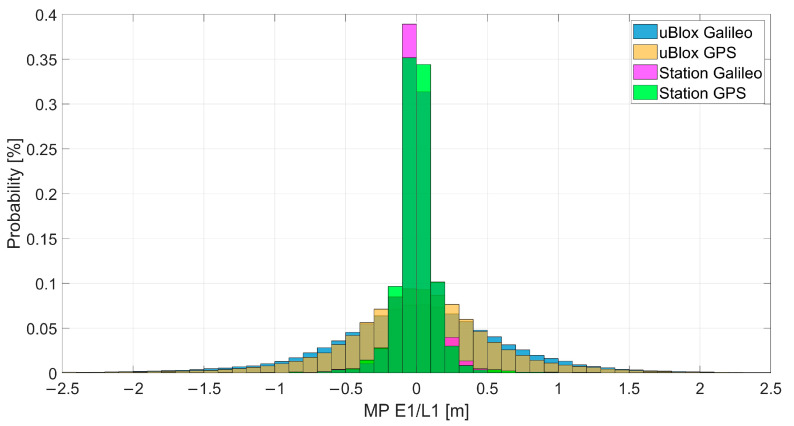
Probability distribution of the multipath errors for E1 and L1, using the U-Blox and professional receiver measurements.

The probability distribution of the multipath errors for the two receivers is shown in Figure 5. From the figure, it can be noted that the multipath error was smaller for the station using the professional receiver, and this was probably due to the setting of the multipath mitigation. For the U-Blox case, no multipath mitigation was used, and larger errors were visible. No big differences can be noted considering Galileo and GPS separately.

## 4. Results

In this section, the experimental results are presented. For the analysis, the HAS corrections were applied to the different available measurements from Galileo and GPS, leading to 14 configurations for the professional receiver case and to 10 configurations for the mass-market receiver.

The configurations considered are showed in Table 1.

For the U-Blox receiver, a subset of configurations was considered because of the available frequencies (see the description in Section 3).

The performance of the different configurations was assessed in terms of mean and root mean square (RMS) error, for 3D, horizontal, and vertical cases. For the vertical error, the absolute value was considered.

**Figure 6:**
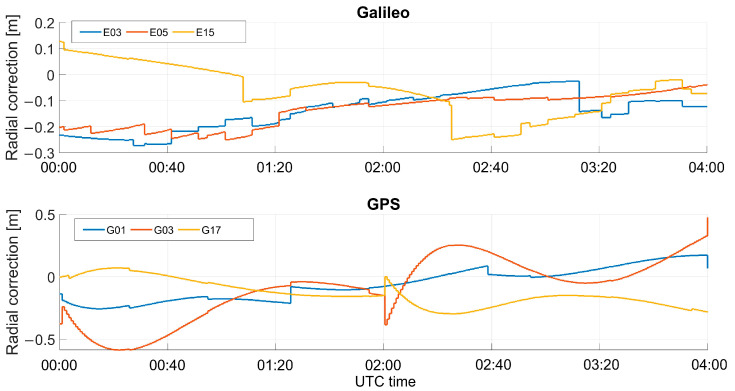
Overview of the applied radial component of HAS orbital correction for three Galileo and GPS satellites.

The orbital corrections, specifically the radial components, applied for some Galileo and GPS satellites, are shown in Figure 6, and only three satellites for each constellation were considered. The corrections were in the order of decimeters: the average radial correction for the Galileo satellite E03 was about −0.1390 m, while for the GPS satellite G01, the mean radial correction was −0.0626 m.

**Figure 7:**
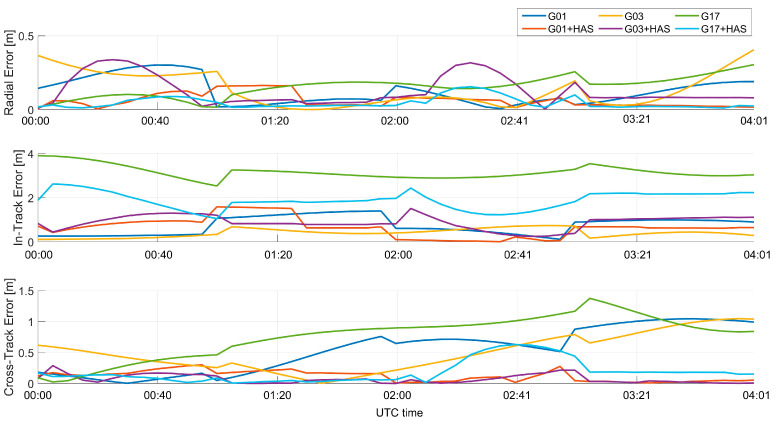
Absolute value of the radial, in-track, and cross-track errors for the GPS satellites with and without HAS corrections.

In order to evaluate the effects of HAS orbital correction on broadcast products, Figure 7 shows the absolute value of the errors, expressed in the NTW frame, of the satellite’s positions computed with the broadcast ephemerides (with and without the application of HAS orbital corrections), with respect to the precise products provided by the NGA (National Geospatial-Intelligence Agency) [31]. As can be seen, differences from a few centimeters to about one meter can be noted. The results are shown only for the GPS case because the impact on Galileo has already been assessed in [12].

### 4.1. Professional Receiver

The results obtained by applying HAS corrections to measurements generated by a professional receiver are discussed in this section.

**Figure 8:**
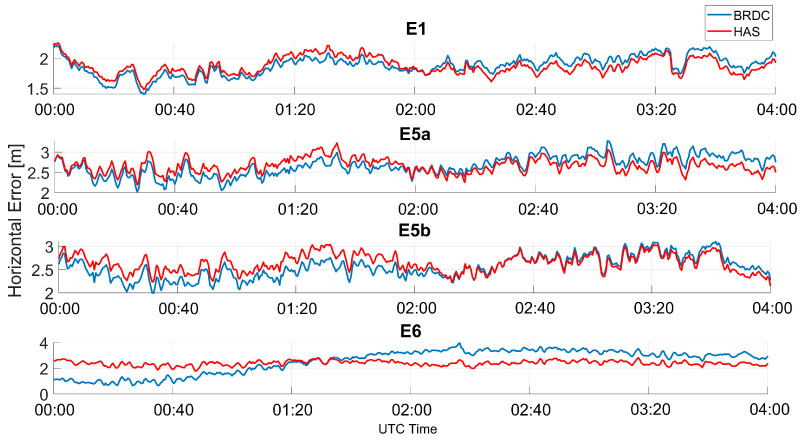
Horizontal error as a function of time for the professional receiver test using only Galileo.

In Figure 8, the horizontal error as a function of time is shown, considering only Galileo solutions; in the upper box, the solutions obtained using E1C measurements are shown, where the blue line represents the case without HAS corrections (referred to as “BRDC”), while the red line represents the solutions obtained by applying HAS corrections (referred to as “HAS”). In each box, a different frequency is considered, and from top to bottom the following frequencies are shown: E1, E5a, E5b, and E6. The color coding described above was adopted for all the frequency cases. For all the frequencies, only small differences between the solution with and without HAS corrections can be noted. In the first part of the data collection, the blue lines were lower than the red lines, while in the second part, the solution computed using the HAS corrections was more accurate. This behavior was more evident for the E6 measurements. In this case, the solution obtained with HAS corrections was flatter while, using only broadcast ephemerides, a small oscillation can be noted. All the solutions show a common trend probably due to the remaining un-modelled errors.

**Figure 9:**
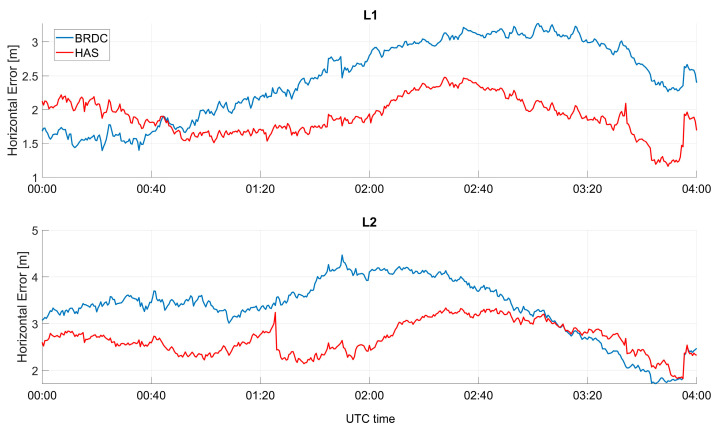
Horizontal error as a function of time for the professional receiver test using only GPS.

In Figure 9, the horizontal error as a function of time is shown, considering only GPS solutions. In the upper box, the solutions using L1C measurements are shown, while in the lower box, the solutions using L2C are considered. Additionally, in this case, the solutions with and without HAS corrections were very close, and a behavior similar to the Galileo-only case could be noted. With respect to the Galileo-only case, the differences were larger, and HAS seemed to strongly reduce the orbit, clock, and measurement errors of the GPS satellites. With respect to the Galileo-only case, the differences between solutions with and without HAS corrections reached about one meter. In addition, the solutions with HAS corrections were flatter than the ones without.

**Figure 10:**
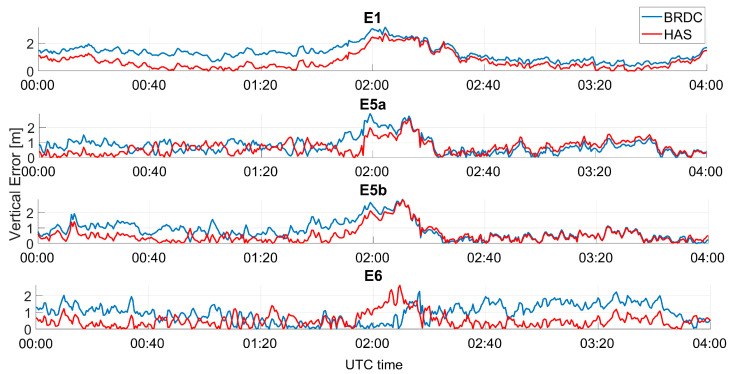
Absolute value of the vertical error as a function of time for the professional receiver test using only Galileo.

**Figure 11:**
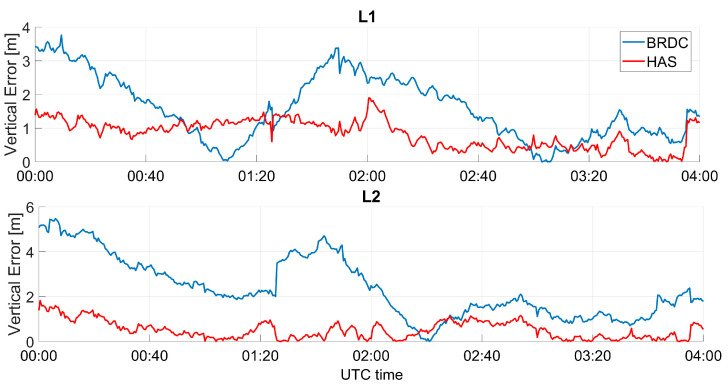
Vertical error as a function of time for the professional receiver test considering GPS only.

In Figure 10 and Figure 11, the absolute value of the vertical errors as a function of time are shown for Galileo and GPS, respectively. In the vertical channel, larger improvements were visible for the GPS-only case, and differences between solutions with and without HAS corrections were of the meter order. The improvements for Galileo-only cases were less visible; however, the errors applying the HAS corrections were lower. The solution using GPS only seemed to be more impacted by the corrections because the baseline solution had a larger error than the Galileo-only cases. Additionally, for the vertical channel, the solutions applying HAS corrections seemed to be flatter, while small oscillations could be noted when using only broadcast ephemerides.

**Figure 12:**
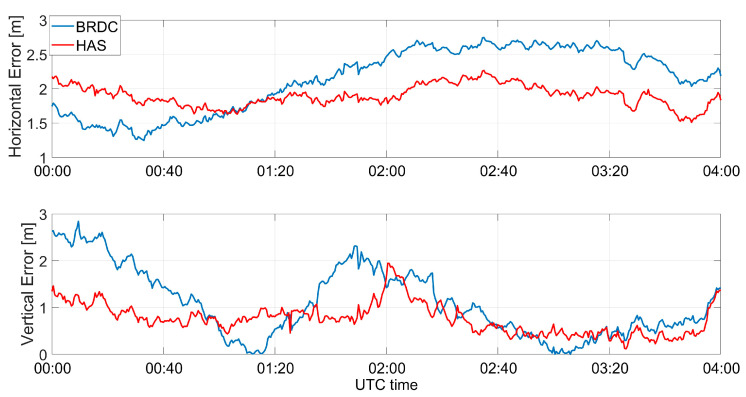
Horizontal and vertical errors as a function of time for the professional receiver test in a multi-constellation solution.

The horizontal and vertical errors of the multi-constellation solutions obtained using Galileo and GPS measurements together are shown in Figure 12. The horizontal and vertical errors are reported in the upper and lower parts, respectively. For the horizontal error, a small increase of the error when using HAS corrections could be noted in the first hour of the test, but the error remained almost constant around 2 m; while for the case without HAS corrections, a larger variation could be noted.

HAS corrections improved the performance of all the considered configurations in terms of mean and RMS error for the horizontal, vertical, and three-dimensional errors. Larger benefits could be appreciated in the vertical channel, where mean and RMS values were below one meter. The statistical parameters of the errors are reported in Table 2.

**Figure 13:**
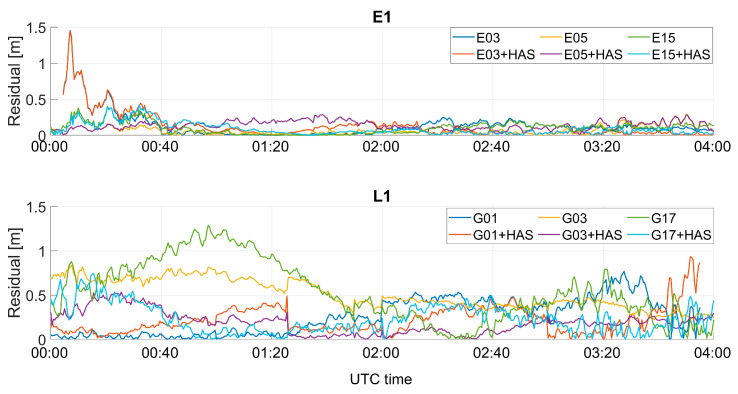
PVT residual analysis for three different Galileo and GPS satellites for the professional test.

Pseudo-range residuals as a function of time for the classical and customized PVT algorithms are shown in Figure 13. For the Galileo case (upper box), the residuals’ difference was smaller than the GPS case. For the GPS case, the largest differences could be noted for the G17 satellite, in the time windows between 00:40 and 02:00. Similar results have been obtained for the pseudo-range residuals on the different frequencies, so to avoid repetition of similar findings, only E1/L1 frequency cases are shown.

### 4.2. Low-Cost Device Test

In this section, the results obtained processing U-Blox data are discussed.

**Figure 14:**
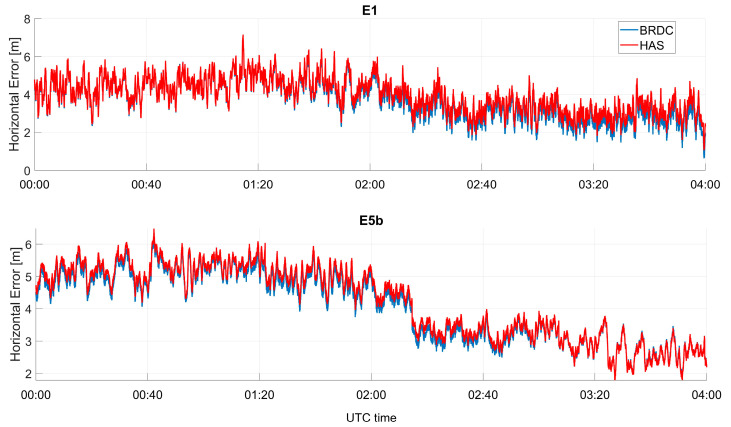
Horizontal error as a function of time for the U-Blox receiver test, considering only Galileo measurements.

**Figure 15:**
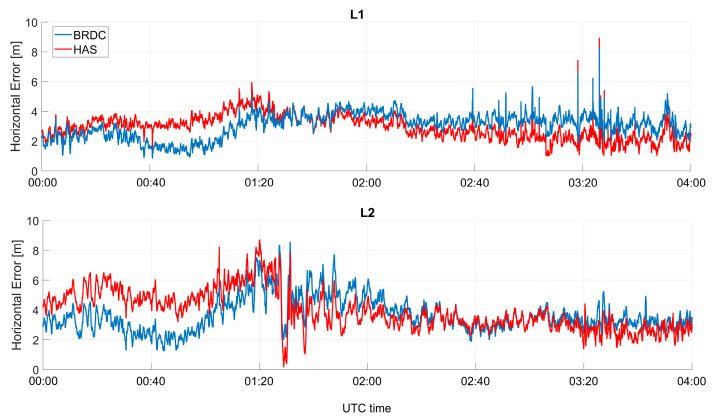
Horizontal error as a function of time for the U-Blox test, considering only GPS measurements.

In Figure 14 and Figure 15, the horizontal errors for Galileo-only and GPS-only cases are shown, respectively. In the upper boxes, the solutions computed using the first frequency (E1C for Galileo and L1C for GPS) are considered, while in the lower boxes, the solutions using the second frequency (E5b for Galileo and L2C for GPS) are shown. For the Galileo case, the solutions with and without HAS corrections were very similar: blue and red lines were almost over-imposed. For the GPS case, larger differences between the solutions with and without HAS corrections could be noted. Additionally, in this case, the horizontal error at the beginning of the test seemed to be larger when HAS corrections were applied, but the error was more stable over time: when only broadcast ephemerides were used, a larger variation of the error was visible.

**Figure 16:**
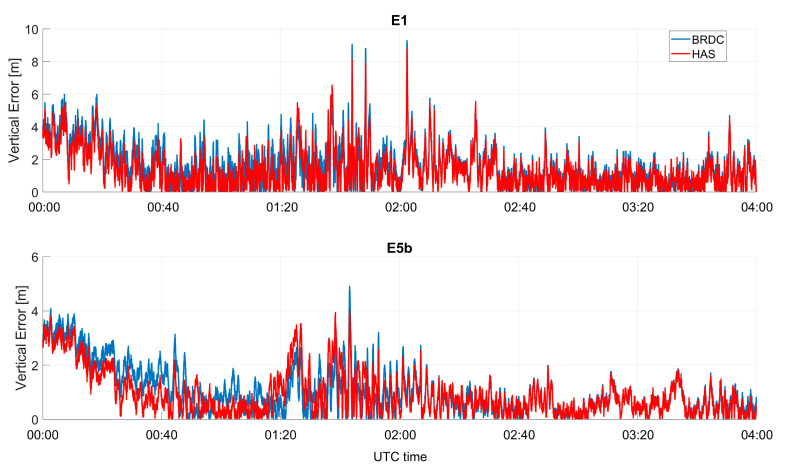
Vertical error as a function of time for the U-Blox test using only Galileo.

**Figure 17:**
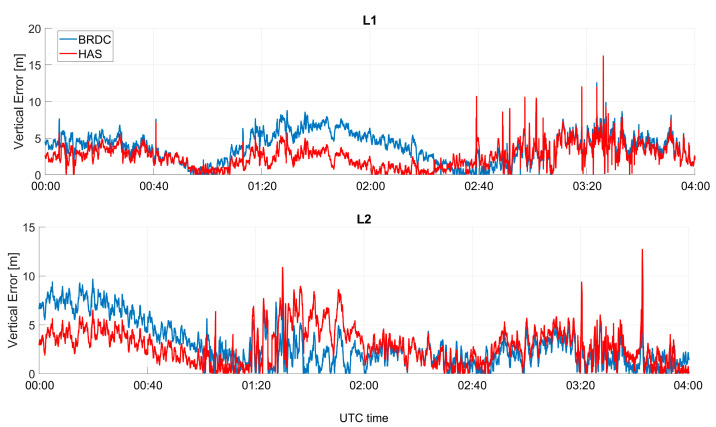
Vertical error as a function of time for the U-Blox test using only GPS measurements.

The vertical errors for Galileo and GPS solutions as a function of time are plotted in Figure 16 and Figure 17, respectively. In all the cases, the errors had similar trends: the larger benefits could be appreciated for the GPS L1C case in the timeframe between 1:00 and 2:30. For Galileo cases, only cm-level differences have been observed during the whole test.

**Figure 18:**
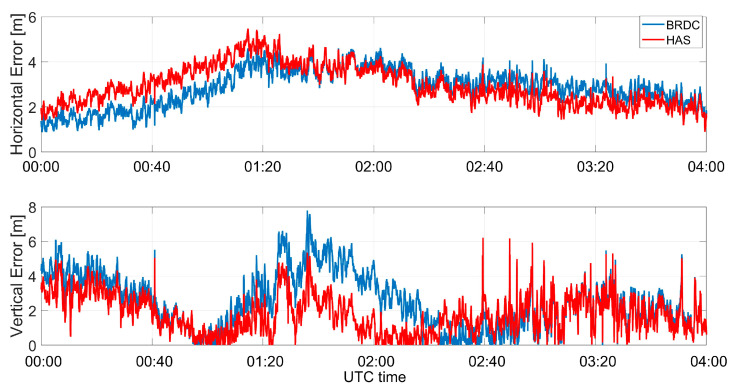
Horizontal and vertical errors as a function of time for the U-Blox device using both Galileo and GPS measurements.

The time evolutions of the horizontal and vertical errors for the multi-constellation solution are shown in Figure 18, in the upper and lower boxes, respectively. Additionally, in this case, a larger horizontal error when using HAS corrections was observed in the first hour of the test, while in the second part of the test, the error using HAS corrections was reduced. The vertical error seemed to be more impacted by the application of the HAS corrections; in this case, the red line was almost always lower than the blue one, with more evidence in the timeframe between 01:20 and 02:20.

The statistical parameters for all the configurations analyzed using the U-Blox receiver are shown in Table 3. From the table, it can be noted that the larger benefits of the application of the HAS correction were in the vertical channel. It is worth noting that the solution using Galileo E5b measurements was the only one with a mean vertical error below one meter (0.92 m).

**Figure 19:**
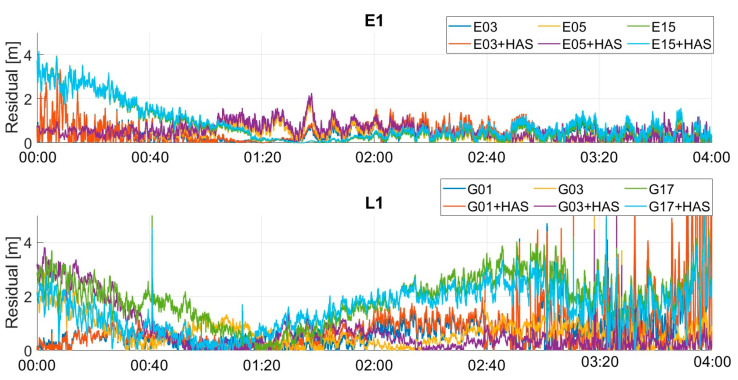
PVT residual analysis for three different Galileo and GPS satellites for the U-Blox test.

The pseudo-range residuals with and without HAS correction are shown in Figure 19. Additionally, for the mass-market receiver case, very small differences could be noted in the Galileo case; indeed, the lines were almost over-imposed. For the GPS case, instead, more evident differences were visible, especially for the G17 satellite, between 00:40 and 01:20 and between 02:40 and 03:00.

## 5. Conclusions

HAS is a novel service developed by Galileo with the aim to enable positioning performance in the order of 20 cm using PPP approaches. Although the service was broadcast for correction for PPP users, the objective of this study was to evaluate the benefits of the service on a simple technique, such as SPP, assessing the enhancements of the computed position. A classical SPP algorithm was modified and tested using real data. Tests were carried out employing two receivers of different grades in static open-sky conditions. About four hours of data were collected and analyzed. The algorithm was able to apply the HAS corrections to all the available frequencies for Galileo (E1, E5a, E5b, and E6) and GPS (L1 and L2) in single- and multi-constellation configurations. For the professional receiver, 14 configurations were tested, while for the mass-market device, only 10 configurations were tested. From the results, it emerged that for the professional receiver case, HAS corrections improved the performance of all the considered configurations in terms of mean and RMS errors for the horizontal, vertical, and three-dimensional errors. Larger benefits have been appreciated in the vertical channel, where mean and RMS values were below one meter. For the mass-market device, it also emerged that the larger benefits of the application of the HAS correction were in the vertical channel. It is worth noting that the solution using Galileo E5b measurements was the only one with a mean vertical error below one meter. For both types of devices, the larger differences between the solutions with and without HAS corrections were observed for the GPS single-constellation case. For all the configurations using HAS corrections, the error was more stable over time. As can be seen from the graphic results, more evident benefits could be noted for the professional receiver. This was probably due to the noisy observations characterizing the low-cost receiver.

The tests analyzed in this work were performed in an open-sky scenario. Additional analyses could address the use of HAS in disturbed environments, in synergy with integrity algorithms.

## Figures and Tables

**Table 1:** List of the adopted configurations.

Configuration	Description	Receivers
**Galileo E1**	Galileo single-constellation solution using E1 with broadcast ephemerides	U-Blox F9P, STONEX SC2200
**Galileo E1 + HAS**	Galileo single-constellation solution using E1 with broadcast ephemerides and HAS correction	U-Blox F9P, STONEX SC2200
**Galileo E5a**	Galileo single-constellation solution using E5a with broadcast ephemerides	STONEX SC2200
**Galileo E5a + HAS**	Galileo single-constellation solution using E5a with broadcast ephemerides and HAS correction	STONEX SC2200
**Galileo E5b**	Galileo single-constellation solution using E5b with broadcast ephemerides	U-Blox F9P, STONEX SC2200
**Galileo E5b + HAS**	Galileo single-constellation solution using E5a with broadcast ephemerides and HAS correction	U-Blox F9P, STONEX SC2200
**Galileo E6**	Galileo single-constellation solution using E6 with broadcast ephemerides	STONEX SC2200
**Galileo E6 + HAS**	Galileo single-constellation solution using E6 with broadcast ephemerides and HAS correction	STONEX SC2200
**GPS L1**	GPS single-constellation solution using L1 with broadcast ephemerides	U-Blox F9P, STONEX SC2200
**GPS L1 + HAS**	GPS single-constellation solution using L1 with broadcast ephemerides and HAS correction	U-Blox F9P, STONEX SC2200
**GPS L2**	GPS single-constellation solution using L2 with broadcast ephemerides	U-Blox F9P, STONEX SC2200
**GPS L2 + HAS**	GPS single-constellation solution using L2 with broadcast ephemerides and HAS correction	U-Blox F9P, STONEX SC2200
**GAL + GPS E1/L1**	Galileo and GPS multi-constellation case using E1/L1 with broadcast ephemerides	U-Blox F9P, STONEX SC2200
**GAL + GPS E1/L1 + HAS**	Galileo and GPS multi-constellation case using E1/L1 with broadcast ephemerides and HAS corrections	U-Blox F9P, STONEX SC2200

**Table 2:** Statistical parameters of the errors: mean and RMS, for horizontal (H), vertical (U), and 3D. Professional receiver cases.

GNSS	Configuration	Mean			RMS		
		H	U	3D	H	U	3D
GAL	E1	1.88	1.31	2.29	1.88	1.43	2.37
	E1 + HAS	1.87	0.72	2.01	1.88	0.95	2.11
	E5a	2.66	0.80	2.78	2.68	0.98	2.84
	E5a + HAS	2.66	0.68	2.75	2.67	0.83	2.80
	E5b	2.55	0.83	2.68	2.56	1.02	2.76
	E5b + HAS	2.64	0.54	2.70	2.65	0.75	2.75
	E6	2.52	0.97	2.71	2.69	1.11	2.91
	E6 + HAS	2.39	0.52	2.45	2.40	0.67	2.49
GPS	L1	2.46	1.60	2.94	2.53	1.86	3.13
	L1 + HAS	1.90	0.83	2.08	1.93	0.93	2.13
	L2	3.33	2.37	4.09	3.40	2.72	4.35
	L2 + HAS	2.68	0.51	2.73	2.7	0.65	2.78
GPS + GAL	L1/E1	2.14	1.08	2.40	2.19	1.31	2.55
	L1/E1 + HAS	1.90	0.76	2.05	1.91	0.83	2.08

**Table 3:** Statistical parameters of the errors: mean and RMS, for horizontal (H), vertical (U), and 3D. U-Blox cases.

GNSS	Configuration	Mean (m)			RMS (m)		
		H	U	3D	H	U	RMS
GAL	E1	3.73	1.63	4.07	3.86	2.07	4.38
	E1 + HAS	3.93	1.36	4.16	4.03	1.78	4.41
	E5b	4.07	1.04	4.20	4.21	1.35	4.42
	E5b + HAS	4.19	0.92	4.29	4.34	1.21	4.51
GPS	L1	3.04	3.78	4.85	3.15	4.27	5.31
	L1 + HAS	2.92	2.54	3.88	3.03	2.97	4.25
	L2	3.66	2.92	4.68	3.83	3.70	5.32
	L2 + HAS	3.93	2.89	4.88	4.15	3.42	5.38
GPS + GAL	L1/E1	2.85	2.51	3.80	2.97	2.96	4.19
	L1/E1 + HAS	2.95	1.80	3.46	3.08	2.09	3.72
